# Shared Segment Analysis and Next-Generation Sequencing Implicates the Retinoic Acid Signaling Pathway in Total Anomalous Pulmonary Venous Return (TAPVR)

**DOI:** 10.1371/journal.pone.0131514

**Published:** 2015-06-29

**Authors:** Dustin Nash, Cammon B. Arrington, Brett J. Kennedy, Mark Yandell, Wilfred Wu, Wenying Zhang, Stephanie Ware, Lynn B. Jorde, Peter J. Gruber, H. Joseph Yost, Neil E. Bowles, Steven B. Bleyl

**Affiliations:** 1 Department of Pediatrics (Division of Cardiology), University of Utah School of Medicine, Salt Lake City, UT, United States of America; 2 Department of Human Genetics, University of Utah, Salt Lake City, UT, United States of America; 3 USTAR Center for Genetic Discovery, Salt Lake City, UT, United States of America; 4 The Heart Institute, Cincinnati Children’s Hospital Medical Center, Cincinnati, OH, United States of America; 5 Cardiothoracic Surgery, University of Utah School of Medicine, Salt Lake City, UT, United States of America; 6 Neurobiology and Anatomy, University of Utah, Salt Lake City, UT, United States of America; 7 Clinical Genetic Institute, Intermountain Healthcare, Salt Lake City, UT, United States of America; Laboratoire de Biologie du Développement de Villefranche-sur-Mer, FRANCE

## Abstract

Most isolated congenital heart defects are thought to be sporadic and are often ascribed to multifactorial mechanisms with poorly understood genetics. Total Anomalous Pulmonary Venous Return (TAPVR) occurs in 1 in 15,000 live-born infants and occurs either in isolation or as part of a syndrome involving aberrant left-right development. Previously, we reported causative links between TAVPR and the *PDGFRA* gene. TAPVR has also been linked to the *ANKRD1/CARP* genes. However, these genes only explain a small fraction of the heritability of the condition. By examination of phased single nucleotide polymorphism genotype data from 5 distantly related TAPVR patients we identified a single 25 cM shared, Identical by Descent genomic segment on the short arm of chromosome 12 shared by 3 of the patients and their obligate-carrier parents. Whole genome sequence (WGS) analysis identified a non-synonymous variant within the shared segment in the *retinol binding protein 5* (*RBP5*) gene. The *RBP5* variant is predicted to be deleterious and is overrepresented in the TAPVR population. Gene expression and functional analysis of the zebrafish orthologue, *rbp7*, supports the notion that *RBP5* is a TAPVR susceptibility gene. Additional sequence analysis also uncovered deleterious variants in genes associated with retinoic acid signaling, including *NODAL* and *retinol dehydrogenase 10*. These data indicate that genetic variation in the retinoic acid signaling pathway confers, in part, susceptibility to TAPVR.

## Introduction

Despite the high frequency with which congenital heart defects (CHD) occur and the great expense to society that these defects incur, the cause of most CHD remains unknown. One such CHD, Total Anomalous Pulmonary Venous Return (TAPVR), occurs in roughly 1 in 15,000 live-born infants [1] and is fatal if not surgically corrected within the first few days to weeks of life. In TAPVR the pulmonary veins connect to the systemic circulation via anomalous vessels instead of to the left atrium. TAPVR can either be an isolated CHD or occur as part of a genetic syndrome, most commonly in the laterality defect spectrum including heterotaxy. Partial anomalous pulmonary venous return (PAPVR) is less severe than TAPVR and occurs when some of the pulmonary veins connect to the left atrium but others connect to the right atrium. Familial clustering of TAPVR has been widely reported, but few candidate genes have been identified to explain the heritability of the defect. We previously reported a founder-effect haplotype on chromosome 4 that segregated through an extended family with TAPVR and localizes to the intergenic region between *PDGFRA* and *KIT*, suggesting a defect in gene regulation [2,3]. TAPVR was observed with reduced penetrance (~7%) in chick and mouse models with loss of function *PDGFRA* mutations [3]. The *ANKRD1/CARP* gene, which encodes a regulator of cardiac transcription, has also been associated with TAPVR after a breakpoint near *ANKRD1* was discovered in a TAPVR patient with a chromosome 10 and21 balanced translocation (t(10;21)) [4]. Together these genes explain only a small fraction of the heritability of the condition.

We hypothesized that a focused search of Identical by Descent (IBD) shared genomic segments (SGS), in distantly related TAPVR patients, could identify deleterious mutations contributing to the heritability of TAPVR. In this study we describe the discovery of an SGS shared by 3 distantly related individuals with TAPVR and the identification of a potential susceptibility variant that is over-represented in TAPVR patients and is involved in retinoic acid signaling.

## Materials and Methods

### Patient identification, enrollment and phenotyping

After approval by the University of Utah Institutional Review Board (Protocol #00021080), we queried the Utah Population Database (UPDB) for all patients diagnosed with TAPVR at Primary Children’s Hospital in order to identify distantly related patients. This cohort consisted of both isolated and laterality-related TAPVR. We selected five TAPVR patients for genotyping due to the presence of multiple shared ancestors. After obtaining informed consent in writing (from parents/guardians for patients less than 18 years of age), peripheral blood samples were obtained from patients and parents. DNA was isolated using a Gentra Autopure LS (Qiagen, Valencia, CA) and analyzed by agarose gel electrophoresis to confirm the integrity of the DNA and quantitated using a Nanodrop.

High-density genotyping was performed on the DNA samples for 1.1 million SNP markers using the Illumina Human Omni1-Quad BeadChip in the Genomics Core at the University of Utah on the five individuals with TAPVR and available parents. 68 control subjects, consisting of 27 individuals without TAPVR and their available parents, were genotyped concurrently. We determined haplotype phase using BEAGLE version 3.3.2 [5] and then analyzed these haplotypes in a pair-wise manner for shared segments that were identical by descent (IBD) using Germline version 1.5.1 [6]. We used 5cM as a cutoff for significant evidence of a shared genomic segment, as we have described previously [7].

### Description of replication cohort

The replication cohort (n = 62) consisted of 47 patients with isolated TAPVR or PAPVR, 4 patients with a sinus venosus atrial septal defect (ASD) and PAPVR, 1 patient with right pulmonary vein atresia, 1 patient with d-transposition of the great arteries (TGA) and PAPVR, and 9 patients with heterotaxy who also had either partial or total anomalous pulmonary venous return.

### Whole Genome Sequencing

Based on the presence of a large shared genomic segment discovered on chromosome 12, we selected two individuals for Whole Genome Sequencing (WGS). Whole-genome sequencing (WGS) was carried out by Complete Genomics Inc., (CGI). Ten ug of DNA was submitted for each sample. Sequence mapping, assembly, and the calling of variants were also completed by CGI. The data provided included variant calls, copy number variation, and structural variation calls, in addition to alignment and coverage files.

### Variant Analysis

The initial analysis was focused on variants that resided within the shared segment and carried out using variant annotation analysis and search tool (VAAST) version 1.0.3 [8]. VAAST compares variant frequency and amino acid substitution data against 1092 background genomes to rank variants according to their likelihood of causing disease. The variants found to be shared within the IBD segment were also analyzed using MutationTaster [9], Polyphen2 [10], and SIFT [11] in order to predict their effect on the coding portions of genes. Concordance between the variants in both the genomes overall, and those confined to the SGS were calculated using cgatools version 1.6.0 build 43.

Identification of the *RBP5* variant prompted a search for variants outside of the SGS in genes with Retinoic Acid Response Elements (RARE) or involved in retinoic acid metabolism and involved in heart development. Custom BASH scripts were used to search the VAAST results for any genes involved in these processes for mutations contributing to the disease process.

### Validation of Variants

Variants identified by WGS were confirmed as follows. PCR primers were designed to amplify variants using the ExonPrimer utility (http://ihg.gsf.de/ihg/ExonPrimer.html) and used to amplify DNA from the patient samples analyzed using Platinum Taq DNA polymerase (Life Technologies) (PCR Primers are described in S1 Table). The PCR product was purified by treating with 4μl of Exo-SAP-IT (Affymetrix, Cleveland, OH) at 37°C for 2 hours and 80°C for 15 minutes. An aliquot of DNA was analyzed by agarose gel electrophoresis and then submitted to the University of Utah DNA sequencing core for Sanger sequence analysis.

For screening of candidate genes in the replication cohort, PCR primers to amplify each coding exon were designed as described above (S2 Table). In addition, primers were designed to the putative RARE [12] in intron 1 of *NODAL* using Primer3Plus (http://www.bioinformatics.nl/cgi-bin/primer3plus/primer3plus.cgi) (S2 Table). All patient DNA samples were amplified in duplicate by PCR using Platinum Taq DNA polymerase. After amplification 0.5 μl of LC Green I (Biofire, Salt Lake City, UT) and 1μl DMSO was added to each 10 μl reaction. The mix was heated at 95°C for 5 minutes and then cooled to 4°C. The PCR products were analyzed on a Lightscanner (Biofire), as previously described.[13–15] Samples giving abnormal curves were treated with 4 μl of Exo-SAP-IT and analyzed by DNA sequencing, as above.

### SNP rs7969705 Genotyping in Heterotaxy Patients

Genotyping for the RBP5 SNP-rs7969705 was performed using TaqMan SNP Genotyping assays (Life Technologies) according to the manufacturer’s instructions. Ten-20ng of DNA was used for PCR amplification, in which samples were denatured at 95°C for 10 minutes followed by 40 cycles of 95°C for 15seconds and 60°C for 1 minute (CFX96 real-time PCR detection system, Bio-Rad). End point data acquisition was performed by two cycles of 60°C for 30 seconds. An allelic discrimination was then performed for each assay to determine the corresponding alleles present in each DNA sample (CFX Manager 3.0, Bio-Rad). A total of 149 samples from Caucasian patients with heterotaxy were genotyped for SNP rs7969705. The heterotaxy patient cohort samples came from 2 different studies approved by the Cincinnati Children’s Hospital IRB. IRB# 2011–2751 is a de-identified sample repository. These samples are deidentified and were obtained under a waiver of consent approved by the IRB. Limited clinical information is available for these samples. IRB#2011–2479 prospectively collects samples and clinical information from patients and families with heterotaxy. Participants provide written consent (and assent, as indicated based on age) to participate in the study and minors are enrolled in the study only if a legal guardian consents. Detailed clinical information was available for 114 subjects whereas 35 were enrolled as “heterotaxy” without more detailed clinical information. Of the 114 subjects with more detailed clinical information, 16 had a family history of heterotaxy or isolated congenital heart defects. Eleven subjects had polysplenia, 27 asplenia, and the remainder had normal spleen or undesignated. With regard to cardiac position, 23 had dextrocardia. Forty-two subjects and vessel abnormalities including 11 with abnormal superior vena cava, 29 with interrupted inferior vena cava, 13 with TAPVR, and 2 with PAPVR. Atrial abnormalities were seen in 39 patients including SA node abnormalities, atrial septal defects, and atrial isomerism. The most common congenital heart defects including ventricular septal defects (n = 23), atrioventricular canal defects (n = 21), double outlet right ventricle (n = 20), single ventricle (n = 11), d-transposition of the great arteries (n = 9) and l-transposition of the great arteries (n = 5).

### Zebrafish strains

Wild-type zebrafish (Danio rerio) embryos were obtained from natural crosses of Oregon AB fish. Embryos were maintained in embryo water between 25 and 28°C and staged according to age (hours post fertilization, hpf) and morphological criteria (Westerfield, 1994) [16]. All work with zebrafish was performed with approval from the University of Utah IACUC committee (animal welfare assurance number A3031-01). Individual adult zebrafish were euthanized by being placed in a solution of tricaine methanesulfonate (MS222). Indications for euthanasia included old age or manifestation of disease or behavioral dysfunction.

### Injection of morpholinos and synthesized mRNA

Morpholinos designed against rbp7a and *spaw* were injected into zebrafish embryos at the 1–4 cell stage. For *rbp7a*, 8 ng/embryo of a translation blocking morpholino (5’-GTTCCACAGAAGCTCACAGGCATTT-3’), were injected. For *spaw*, 8 ng/embryo of splice-blocking morpholino (5’-GCACGCTATGACTGGCTGCATTGCG-3’) were injected. In subthreshold experiments, 2 ng/embryo of *Rbp7a* and 1 ng/embryo of *Spaw* morpholinos were injected. Capped sense *rbp7a* and human *rbp5* mRNA were synthesized using the mMessage mMachine in vitro transcription kit (Ambion) and purified as described previously (Rupp et al., 1994). For mRNA rescue experiments a range of 50–200 pg/embryo was injected into the embryo. All morpholino and mRNA injections were repeated at least three times.

### *In situ* hybridization

Digoxigenin-labeled antisense RNA probes were synthesized by in vitro transcription using T3, T7 and SP6 polymerases. cDNA templates used in this study included *rbp7a*, *cmlc2* (Yelon et al., 1999), and spaw (Long et al., 2003). Whole mount *in situ* hybridization was carried out as described previously (Thisse et al., 1993) using a Biolane HTI in situ machine (Huller and Huttner AG). Zebrafish embryos were cleared in 70% glycerol in PBST. Embryos were photographed with a Leica MZ12 stereo microscope using a Dage-MTI DC330 CCD camera.

### Immunohistochemistry

Embryos were fixed overnight in a 50:50 mixture of 8% paraformaldehyde: 1X PBS at 4°C and dehydrated stepwise into methanol for storage at -20°C. After stepwise rehydration, embryos were blocked for 1 hour in PBDT (1% BSA, 1% DMSO, 0.1% Triton X-100 in PBS pH 7.3) prior to treatment with primary or secondary antibodies. Primary antibodies included: 1:100 rabbit polyclonal anti-ζPKC (Santa Cruz Biotechnology) and 1:200 mouse IgG anti-acetylated tubulin (Sigma). Alexa Fluor antibodies (Molecular Probes) were used as secondary antibodies at a concentration of 1:200. Dissected embryos were equilibrated in Slowfade Anti-fade buffer (Molecular Probes) and mounted on microscope slides for KV imaging. Embryos were imaged using an Olympus Fluoview scanning laser confocal microscope with a 60X objective. To ensure consistent adjustment of microscope power and gain settings, wild-type embryos were imaged in all experiments.

### Statistical Analyses

Comparisons of genotypes between patient populations and controls were performed using chi-square analysis. Comparisons of end-points in zebrafish studies were performed by Student's t-test. In all cases a P value <0.05 was considered significant.

## Results

### Identity by descent mapping

**Fig 1:**
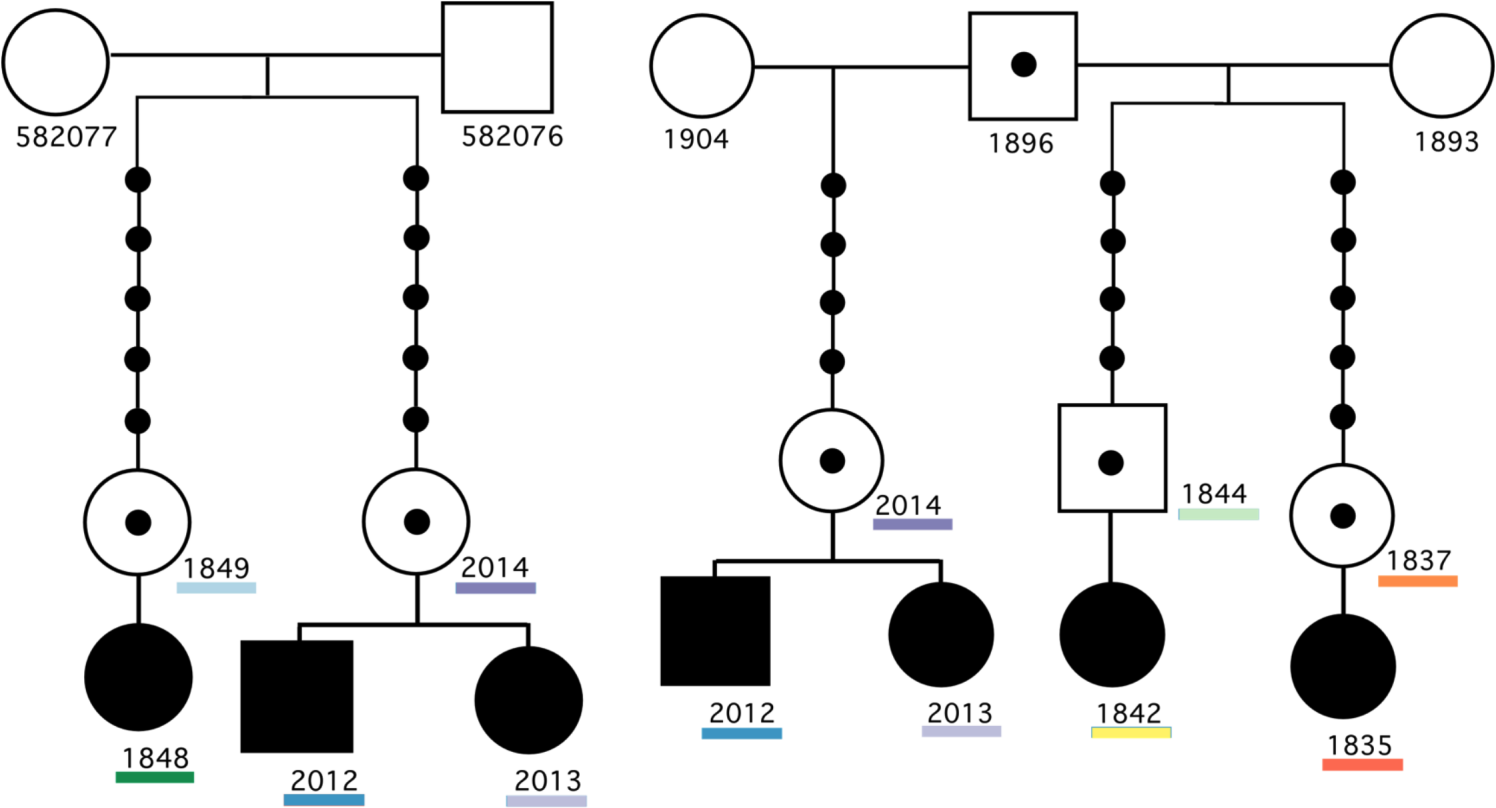
Identification of Shared Genomic Segments in TAPVR pedigrees. Two pedigrees with TAPVR affected patients which shared genomic segments greater than 5cM. The colors below the patient ID correspond with the IBD segments shown in Fig 2. Note, the affected siblings 2012 and 2013 are related to other TAPVR affected individuals through two separate lineages (both through their mother) as depicted by two pedigrees.

Using the Utah Population Database we identified 5 patients with TAPVR who are related through multiple founder individuals (Fig 1). All patients are separated by at least 11 meioses, with the exception of two affected siblings. Despite knowing of the shared ancestors, it was not possible to determine *a priori* which, if any, of these relationships were related to the occurrence of TAPVR. We therefore pursued a pair-wise analysis to detect any shared genomic segments that were inherited from a common ancestor.

**Fig 2:**
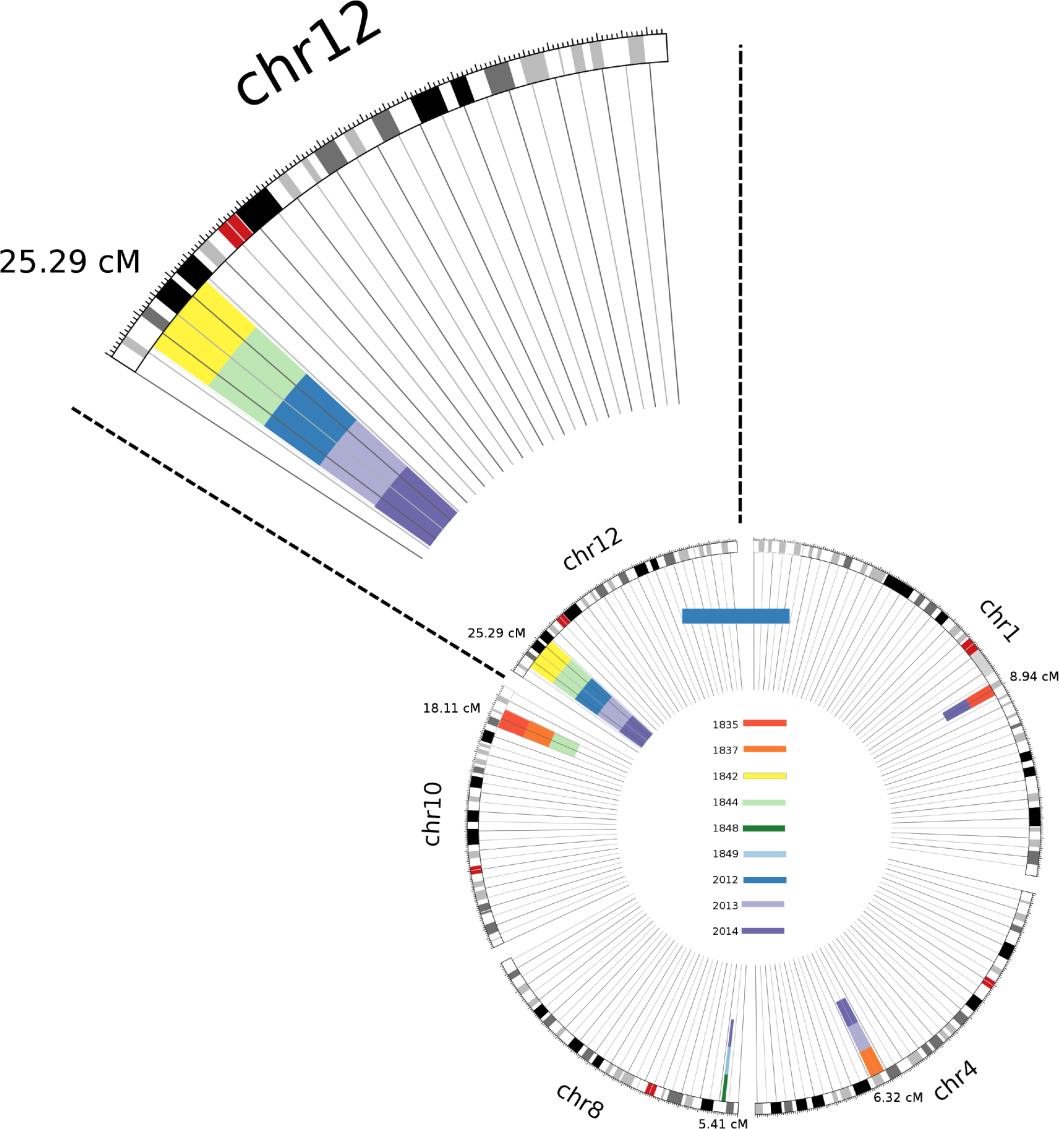
Representation of the IBD segments >5cM that were shared by 2 or more TAPVR patients or their parents. The different colors on the inside of each circle show the patients which shared the IBD segment at each of the positions (based in the color code in Fig 1).

Using Germline software [6] we compared SNP array data in a pairwise fashion to identify any genomic sharing among affected individuals. Six shared genomic segments (SGS) greater than 5cM in length were identified among the cohort, one of which was a 25.3 cM segment on the short arm of chromosome 12 (Fig 2). This segment is shared by two affected siblings (patients 2012 and 2013) and a distantly related cousin (1842) and inherited though obligate-carrier parents (2014 and 1844). Haplotype analysis of the shared segment revealed that it extends from 6,520,956bp to 24,198,980bp (hg19).

### Whole genome sequencing (WGS) and variant analysis

Patients 2012 and 1842 were selected for next generation sequencing to look for damaging variants within the SGS that might confer a susceptibility to TAPVR. Concordant and damaging genetic variants were obtained by performing VAAST analysis [8] of all variants in both genomes. From this analysis, variants in 3939 genes were identified as potentially deleterious. Of these, 30 variants in 23 genes were shared within the SGS on chromosome 12 but none reached genome-wide statistical significance (Table 1). Of these variants, 17 were exonic while 6 were found in non-coding elements. MutationTaster analysis [9] predicted 7 to be damaging, of these 4 were exonic and 3 were in non-coding regions. Both Polyphen2 [10] and SIFT [11], which only analyze non-synonymous coding changes, predicted that 4/23 were deleterious (Table 2). Only the gene variant in *RBP5* (c.208G>C, p.Glu70Gln: rs7969705) was predicted by all *in silico* methods to be disease causing/deleterious. The affected glutamic acid is located in a lipocalin/cytosolic fatty-acid binding domain. Transmission of this variant through individuals 2012, 2013, 2014, and 1842 was confirmed by Sanger sequencing.

**Table 1:** VAAST Analysis of the WGS variants identified in the chromosome 12 SGS.

RANK	Gene	p-value	Score	Position	Base Change	Amino Acid Substitution	Variant Frequencies (Background)	Variant Frequencies (Target)
2	NANOG	1.30E-04	40.97397084	7948160	A->G	N/A	11	4
4	BCL2L14	7.64E-04	15.305536	12247720	T->C	N/A	0	2
7	STRAP	1.90E-03	15.305536	16056203	C->T	N/A	0	2
8	TAPBPL	4.17E-03	19.25459877	6562823	C->T	A->V	567	4
9	SCNN1A	7.25E-03	15.305536	6456189	G->A	N/A	0	2
10	VAMP1	8.73E-03	7.45447321	6572587	G->C	N/A	499	4
14	LRRC23	4.00E-02	12.42527828	7022085	T->A	V->E	850	4
20	CDKN1B	5.93E-02	4.8906837	12870572	C->T	N/A	9	2
21	CLEC1B	6.20E-02	3.56272311	10150974	A->G	S->P	916	4
22	LOH12CR1	6.87E-02	3.09117152	12618690	G->A	D->N	208	2
23	PIK3C2G	7.86E-02	7.80486734	18435452	C->T	P->L	766	4
24	PRB4	8.43E-02	9.38745315	11461802	G->A	R->*	221	2
25	PRH2	9.29E-02	0.36282216	11083356	A->G	N->D	528	3
27	LST-3TM12	1.19E-01	4.61687526	21207389	T->C	F->L	193	2
28	SLCO1B1	1.20E-01	2.11035239	21329813	C->A	P->T	174	2
30	SLCO1B3	1.39E-01	1.23770967	21028208	G->C	G->A	175	2
34	RBP5	1.57E-01	1.99092931	7280880	C->G	E->Q	163	2
39	TAS2R31	2.10E-01	3.86997817	11183832	G->A	R->W	874	3
41	CLEC2D_DUP_01	2.30E-01	0.465915	9822387	C->G	N->K	444	2
46	ATF7IP	3.27E-01	1.12294062	14577892	A->T	N->I	705	3
49	SLCO1C1	4.03E-01	0.45116847	20905250	C->T	S->F	1069	3
51	PZP	4.67E-01	0.82759961	9303296	A->T	I->N	446	2
53	TAS2R19	6.10E-01	0.58069098	11174276	G->A	R->^	1099	3

**Table 2:** Variants in the chromosome 12 SGS predicted damaging/deleterious by MutationTaster/Polyphen2 and SIFT.

	Position	Gene	Reference->Variant
MutationTaster			
	12:6887020	LAG3	T->C
	12:7280880	RBP5	C->G
	12:6931001	GPR162	>G
	12:8693420	CLEC4E	>GAGA
	12:12510074	LOH12CR1	C->G
	12:12870572	CDKN1B	C->T
	12:14656768	PLBD	C->G
Polyphen2			
	12:11339020	TAS2R42	T->A
	12:12618690	LOH12CR1	G->A
	12:21028208	SLCO1B3	G->C
	12:7280880	RBP5	C->G
SIFT			
	12:6887020	LAG3	T->C
	12:7280880	RBP5	C->G
	12,:16397734	SLC15A5	C->A
	12:11174276	TAS2R19	G->A

The variant is found in the population with a minor allele frequency around 5–9% in Caucasians. To assess whether rs7969705 is overrepresented in our TAPVR cohort, we compared the minor allele (C) frequency in 62 unrelated TAPVR patients to that of race and ethnically matched individuals from the Exome Aggregation Consortium (ExAC) database, Cambridge, MA (URL: http://exac.broadinstitute.org: [May 2015, accessed]). The RBP5 E70Q variant was identified in 15 patients (13 heterozygous and 2 homozygous). As shown in Table 3, the frequency of the G allele in TAPVR patients was found to be roughly twice that of controls, a statistically significant difference when compared using a Chi-square analysis. To further investigate whether this allele is specific to isolated TAPVR, we genotyped rs7969705 in a heterotaxy cohort. Although heterotaxy can be associated with TAPVR, its developmental, genetic, and mechanistic relationship to TAPVR is unclear. The RBP5 E70Q variant was identified in 20 alleles, 18 heterozygous and 2 homozygous, in a cohort of 149 Caucasian heterotaxy patients (Table 3), which was not significantly different from control populations. These results demonstrate that the variant is specifically overrepresented in isolated TAPVR.

**Table 3:** Comparison of the allelic frequency of rs7969705 in 64 TAPVR cases and 149 heterotaxy cases versus non-Finnish Europeans in the ExAC database.

Comparison	Allele 1 (C)	Allele 2 (G)	Marginal Row Values	Chi-square Statistic	Chi-Square P Value
TAPVR vs ExAC				7.54	0.006
	17	107	128		
	5232	66536	71768		
Heterotaxy vs ExAC				0.15	0.701
	20	278	298		
	5232	66536	71768		

### Examination of other genomic variants in the family

Having identified the *RBP5* rs7969705 variant as the likely chromosome 12-linked TAPVR susceptibility but given the fact that the shared *RBP5* variant was passed down through multiple unaffected people, we reasoned that it is a low-risk susceptibility gene and that additional damaging variants, potentially within the retinoic acid pathway, would be found in the TAPVR patients with this variant. In patient 2012, we identified a rare missense variant in the *NODAL* gene that was predicted to be deleterious by Polyphen2, MutationTaster and SIFT. The c.904C>T change (rs150819707; p.Arg302Cys) was also predicted to be significant by VAAST [8] with a genome-wide ranking of 967 (p-value of 0.00826). This variant is rare, having been observed in only 0.08% of non-Finnish Europeans in the ExAC database. The variant was also carried by the patient’s affected sibling (patient 2013) but was not detected in DNA isolated from peripheral blood from their mother. Therefore, it likely comes from the father (DNA not available) although it is possible that the mother was a gonadal mosaic. Patient 1848 was found to carry a rare missense variant in *RDH10* (retinol dehydrogenase 10) that is predicted to be deleterious by Polyphen2 and MutationTaster but not SIFT. This variant (c.322T>G; p.Cys108Gly: rs145171413) is present in 0.8% of Non-Finnish Europeans in the ExAC database. This variant was inherited from the father, whereas the *RBP5* variant was inherited from the mother. Therefore, in both cases, these ‘second hit’ mutations were likely inherited from the parent not carrying the *RBP5* variant, suggesting digenic inheritance.

To further explore a relationship between *RBP5* and *NODAL* variants in TAVPR and PAPVR patients, we screened the entire coding sequences of *RBP5* and *NODAL* by high resolution melting and direct DNA sequencing in our cohort of 62 unrelated TAPVR patients. No additional novel variants were identified in *RBP5*, but we identified a novel missense variant in *NODAL* (c. 742C>T, p.R248W) in a patient with isolated TAPVR and a 2bp exonic insertion (c.318_319insAG: p.Gly107Argfs*24) in patient with laterality defects including TAPVR: the latter was also carried by an affected sibling. The missense variant was predicted to be damaging/deleterious and is not present in the ExAC database. In both cases the *NODAL* variants were inherited from healthy parents.

### Expression and functional analysis of an *RBP5* orthologue in zebrafish

The deleterious nature of the *RBP5* variant is supported on other grounds as well. RBPs regulate the availability of Vitamin A (retinol) in an organism and are important for retinoid acid signaling, a process known to be involved in heart development including patterning of the second heart field. We chose to conduct these studies in zebrafish, a model organism that has been widely used for analysis of cardiac development [17]. We compared the amino acid sequence of the human *RBP5* gene to zebrafish retinol binding proteins and found the highest identity to be 51% in zebrafish rbp7a (S1 Fig), thus positing that *rbp7a* is the *RBP5* orthologue in zebrafish.

**Fig 3:**
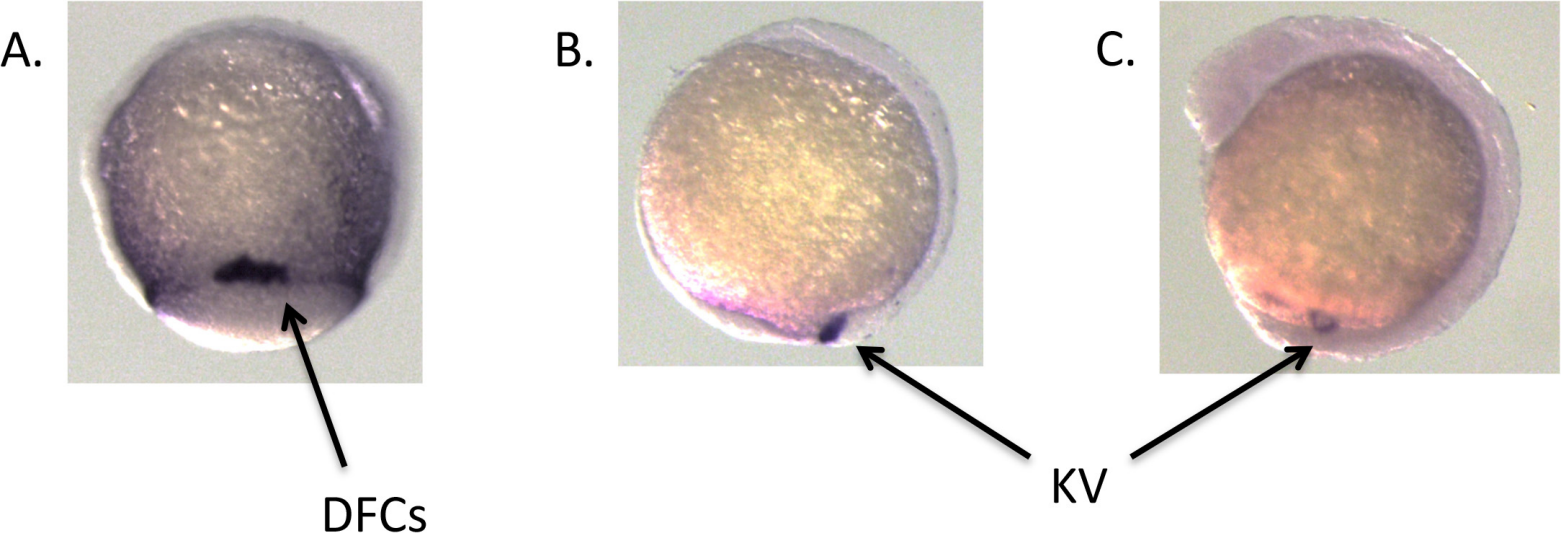
*rbp7a* expression in the developing Zebrafish. *rbp7a* is strongly expressed within the dorsal forerunner cells (DFCs) and Kupffer’s vesicle (KV) in the developing zebrafish embryo: Panel A. 75% epiboly; Panel B. tailbud stage; Panel C. 8 somite stage.

We examined the expression and function of the *rbp7a* gene in the zebrafish model. In early embryogenesis, *rbp7a* expression is strong within the dorsal forerunner cells (DFCs) and the fluid-filled Kupffer’s vesicle (KV) (Fig 3). In zebrafish, the DFCs are a collection of approximately two-dozen mesenchymal cells that migrate at the leading edge of the embryonic shield during gastrulation. At the end of gastrulation, they transition from mesenchymal to epithelial cells to form the ciliated lining of KV. Within the KV, cilia move in a coordinated fashion to generate counterclockwise fluid flow. This process is critical in the establishment of left-right asymmetry and subsequently leads to expression of the Nodal family member *southpaw* (*spaw*) in the left lateral plate mesoderm. Although it is not fully understood how asymmetric fluid flow within KV leads to expression of *spaw* in the lateral plate mesoderm, it has been shown that retinoic acid (RA) signaling is critical for setting up the left-sided Nodal signal through regulation of cilia length and KV fluid flow [18].

**Fig 4:**
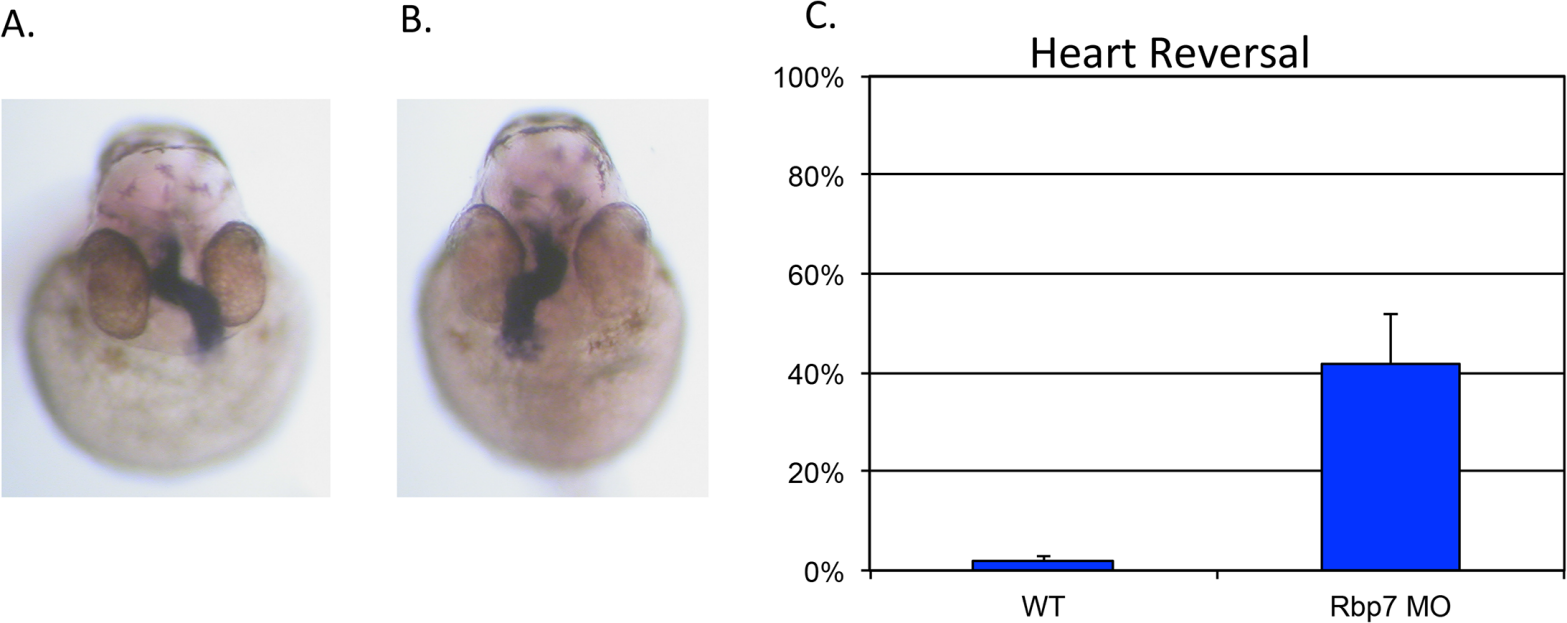
Effects of rbp7a knockdown on zebrafish heart looping. Morpholino knockdown of rbp7a results in randomized heart looping. *cmlc2* expression at 38 hpf is used to visualize heart looping. Panel A: normal, left-sided heart looping; Panel B: reversed, right-sided heart looping; Panel C: Reversed heart looping occurs in 2% of uninjected control (WT) embryos (n = 315) and 42% of *rbp7a* morphant (MO) embryos (n = 312).

**Fig 5:**
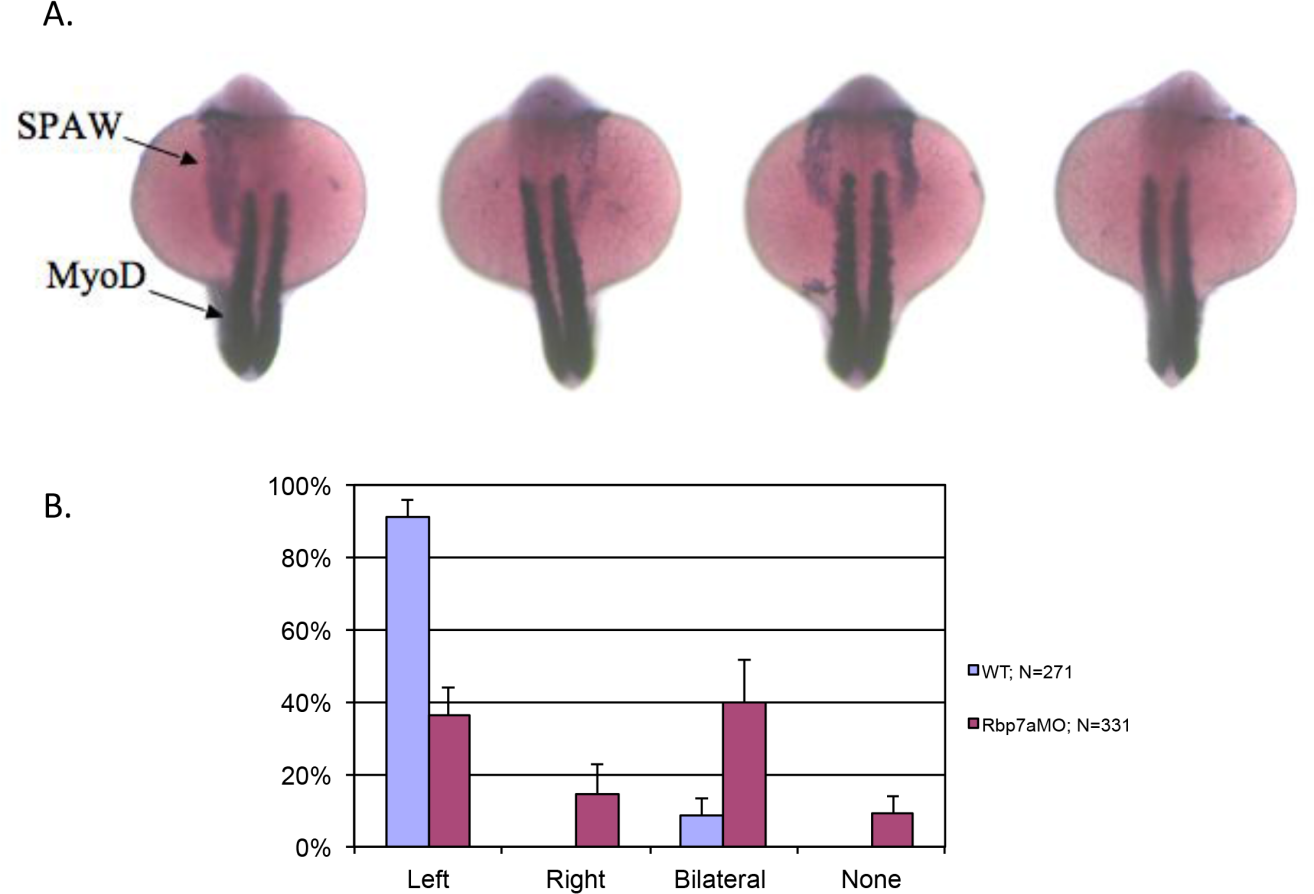
Morpholino knockdown of rbp7a results in perturbed *spaw* expression. Panel A: representative images showing, left-sided *spaw* expression, right sided *spaw* expression, bilateral *spaw* expression and no *spaw* expression. Panel B. *spaw* scoring in WT (blue bars) and *rbp7a* morphant (MO) embryos (purple bars).

To determine whether rbp7a plays a role in left-right patterning, morpholino knockdown experiments were performed. Although the overall appearance of *rbp7a* morphants was normal, morphants displayed reversed heart looping (42% with abnormal right-sided heart looping, n = 312; Fig 4) and aberrant *spaw* expression (only 36% normal left-sided spaw expression, n = 331; Fig 5). Attempts to rescue the laterality phenotype by co-injection of mopholino-resistant rbp7a mRNA as well as human rbp5 mRNA were unsuccessful but not surprising since overexpression of these RNAs also leads to laterality defects (data not shown), suggesting that just the right amount of retinoic acid is required for normal left-right development.

**Fig 6:**
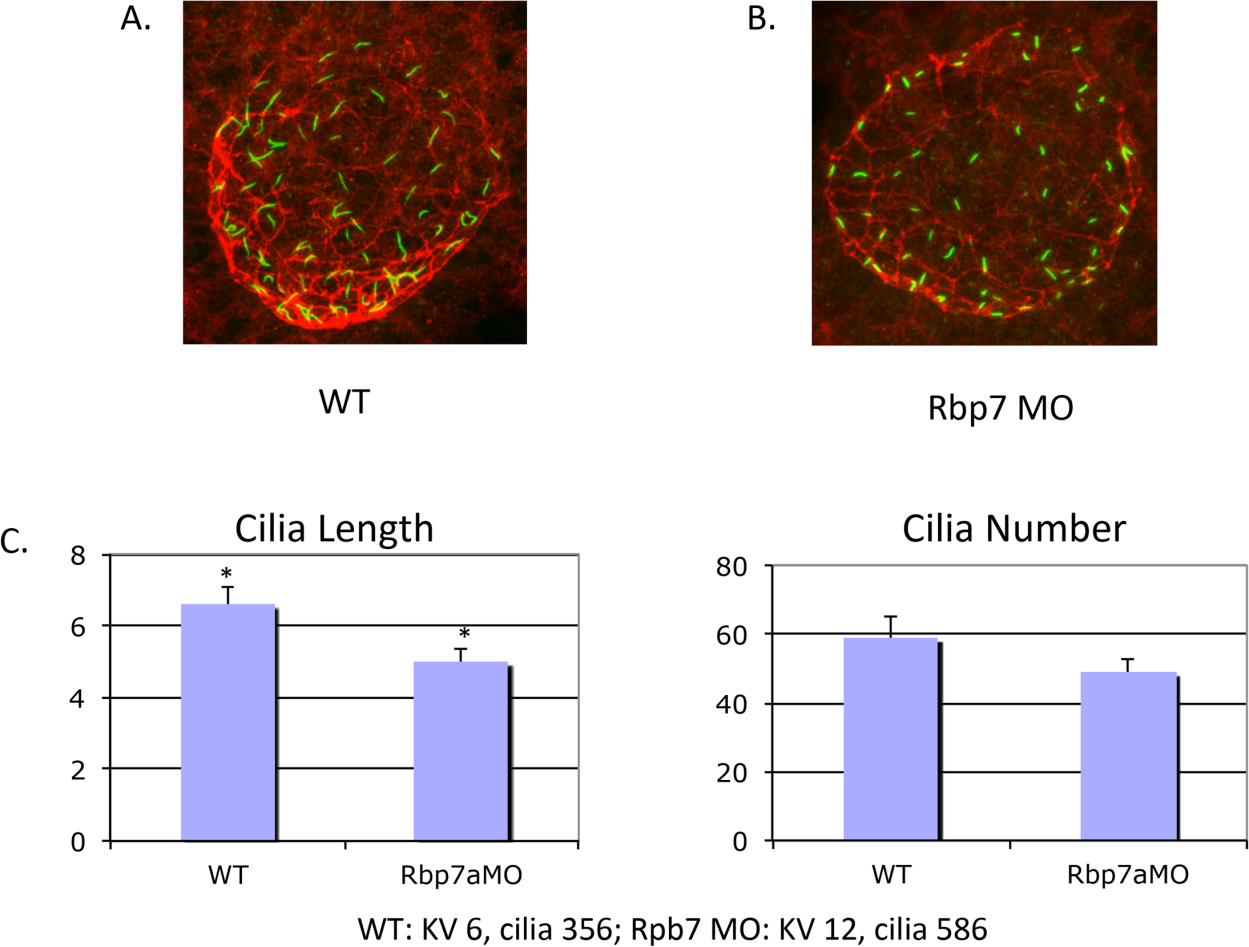
KV cilia are shortened in *rbp7a* morphants. Panels A and B: Immunohistochemical staining of representative WT and morphant KV and cilia. Panel C: *rbp7a* morphants have shorter but not fewer KV cilia than WT embryos. * p<0.05.

We next investigated whether rbp7a is required for KV or cilia formation. KV morphogenesis was normal as visualized by the epithelial marker atypical PKC (anti-ζPKC). However, we found that KV cilia are shorter in *rbp7a* morphants than in WT embryos (Fig 6). The effect of rbp7a on KV cilia in combination with a potential role for rbp7a and retinoic acid in transmitting the laterality signal from the KV to the LPM are likely the reason for aberrant downstream LR development in zebrafish *rbp7a* morphants.

**Fig 7:**
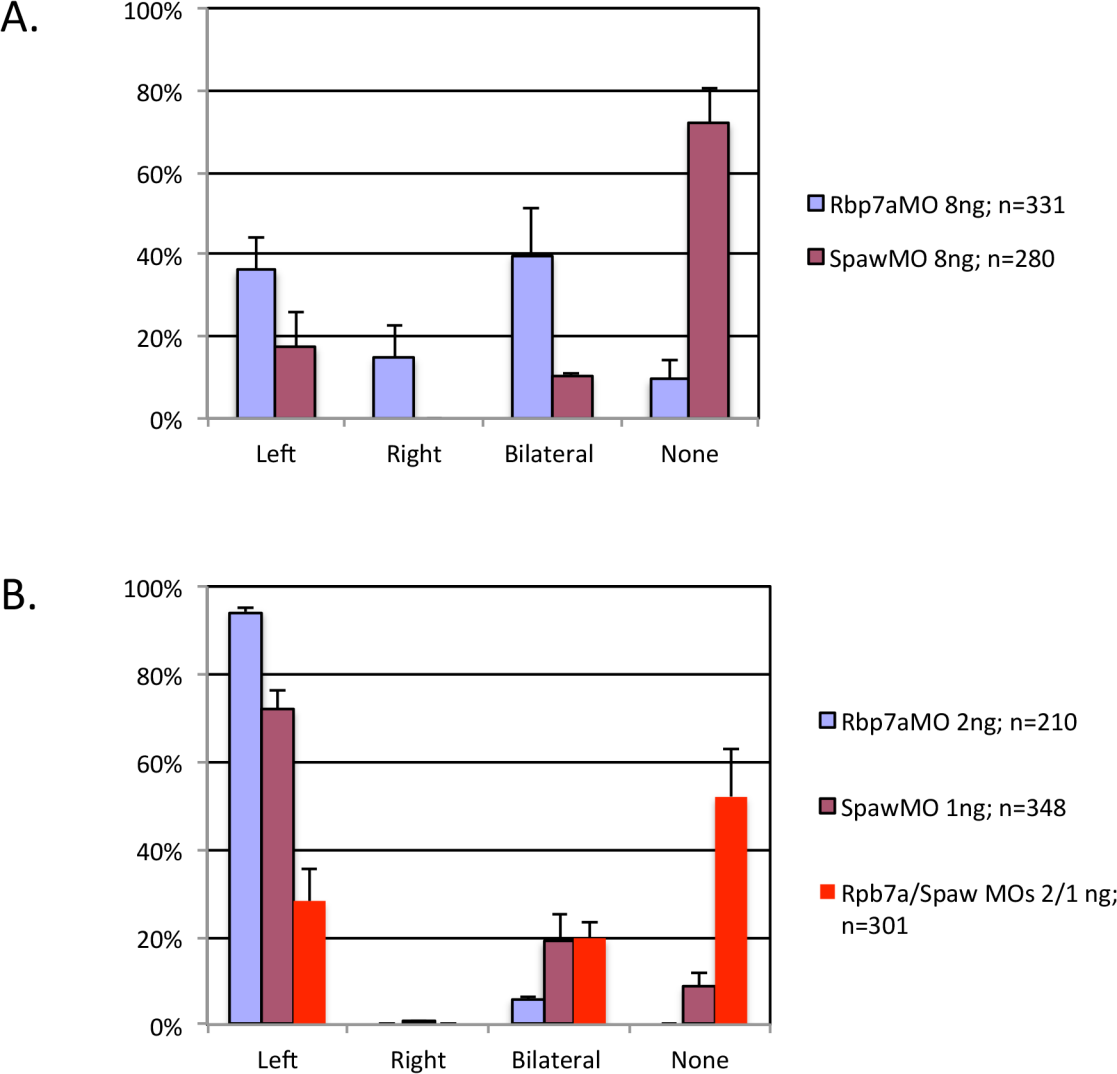
*rbp7a* and *spaw* function together in left-right development. Panel A: Injection of *rbp7a* (blue bars) or *spaw* (purple bars) morpholinos at standard concentrations results in significantly altered *spaw* expression. Panel B: Injection of either morpholino at subthreshold doses (*rbp7a*: blue bars; *spaw*: purple bars) results in more normal left-sided *spaw* expression. However, co-injection of both morpholinos at subthreshold doses leads to significantly altered *spaw* expression, similar to the higher dose *spaw* morphants.

As discussed previously, we discovered other genes with deleterious variants that might combine with RBP5 E70Q to cause pulmonary vein anomalies. In patient 2012 we found a variant in the gene *NODAL* that is predicted to be pathogenic. To determine whether RBP5 and NODAL function within the same developmental pathway, we performed synergy experiments in zebrafish using subthreshold doses of each morpholino. Injection of 2 ng *rbp7a* MO alone or 1 ng *spaw* MO alone did little to perturb LR patterning, as assayed by *spaw* expression in the LPM (Fig 7). In contrast, coinjection of both morpholinos at these subthreshold doses resulted in significantly altered *spaw* expression (Fig 7), with most embryos having absent *spaw*, similar to what is seen with injection of higher doses of *spaw* MO alone. These results indicate that exposure to low dose *rbp7a* MO enhances the effect of *spaw* MO suggesting that these genes act together in left-right development.

## Discussion

By leveraging the ability of the Utah Population Database (UPDB) to identify distantly related TAPVR patients and combining IBD mapping with next-generations sequencing, we have pinpointed a TAPVR-associated gene variant on chromosome 12. The damaging variant in retinol binding protein 5 (*RBP5*) did not reach genome-wide significance in our whole-genome analysis (including VAAST analysis) so without the shared segment mapping that is available through the use of the UPDB, it is unlikely we would have identified this variant as a candidate susceptibility for TAPVR. RBP5 is a retinol binding protein that regulates the levels of retinol within the cell. Retinoic acid signaling is known to be involved in development of the secondary heart field [19,20] from which the pulmonary veins arise [21]. Indeed, animals deficient in retinol during development have structural heart defects affecting the inflow tract where the pulmonary veins develop [19,22].

In addition to the deleterious variant in *RBP5* we found other damaging variants in genes associated with the retinoic acid pathway [19,23]. Patient 1842 carries a rare damaging variant in *RDH10*, a gene involved in retinoic acid metabolism. Siblings 2012 and 2013 harbor variants in *NODAL*, a gene regulated by retinoic acid and involved in left-right patterning. Deleterious *NODAL* variants were also discovered in three other patients with TAPVR (one with isolated TAPVR and two siblings with laterality-related TAPVR). In prior studies, *NODAL* has only been associated with syndromic (laterality-related) TAPVR but our results indicate that both isolated and syndromic forms of anomalous pulmonary venous return have overlapping genetic underpinnings. Although a causative link between these variants and TAPVR will require further validation, our analysis demonstrates the utility of pathway analysis starting from a smaller set of candidate variants in an SGS to better understand the genetics of multifactorial diseases. Links between NODAL expression and retinoic acid expression have already been established. Uehara and colleagues [12] created mice lacking CYP26, the enzyme that degrades retinaldehyde dehydrogenase and is critical in controlling levels of retinoic acid metabolites. These mouse embryos showed complete or partial duplication of the body axis and duplication of the primitive streak, with abnormal expression of *nodal*, similar to mice lacking the nodal antagonists Lefty1 and Cer1. In addition, these authors identified a retinoic acid-responsive element (RARE) in intron 1 that is highly conserved among mammals.

These studies illustrate the shortcomings of strategies that filter on minor allele frequency (i.e., MAF < 0.1) or the use of variant algorithms that emphasize rare variants in the search for disease causing variants for complex, multifactorial diseases where some contributing factors may not be particularly rare. In fact, we would argue that the use of MAF cutoffs in the analysis of complex conditions like congenital cardiac malformations is difficult to defend. We have provided significant evidence for the involvement of a relatively common *RBP5* variant in TAPVR, a variant that did not meet genome-wide significance by typical variant analysis and which would have been eliminated using most MAF-based filtering schemes. We further contend that variants such as *RBP5* rs7969705 that are overrepresented in disease states and involved in critical signaling pathways, would be high-value targets in the study of multifactorial disease.

**Fig 8:**
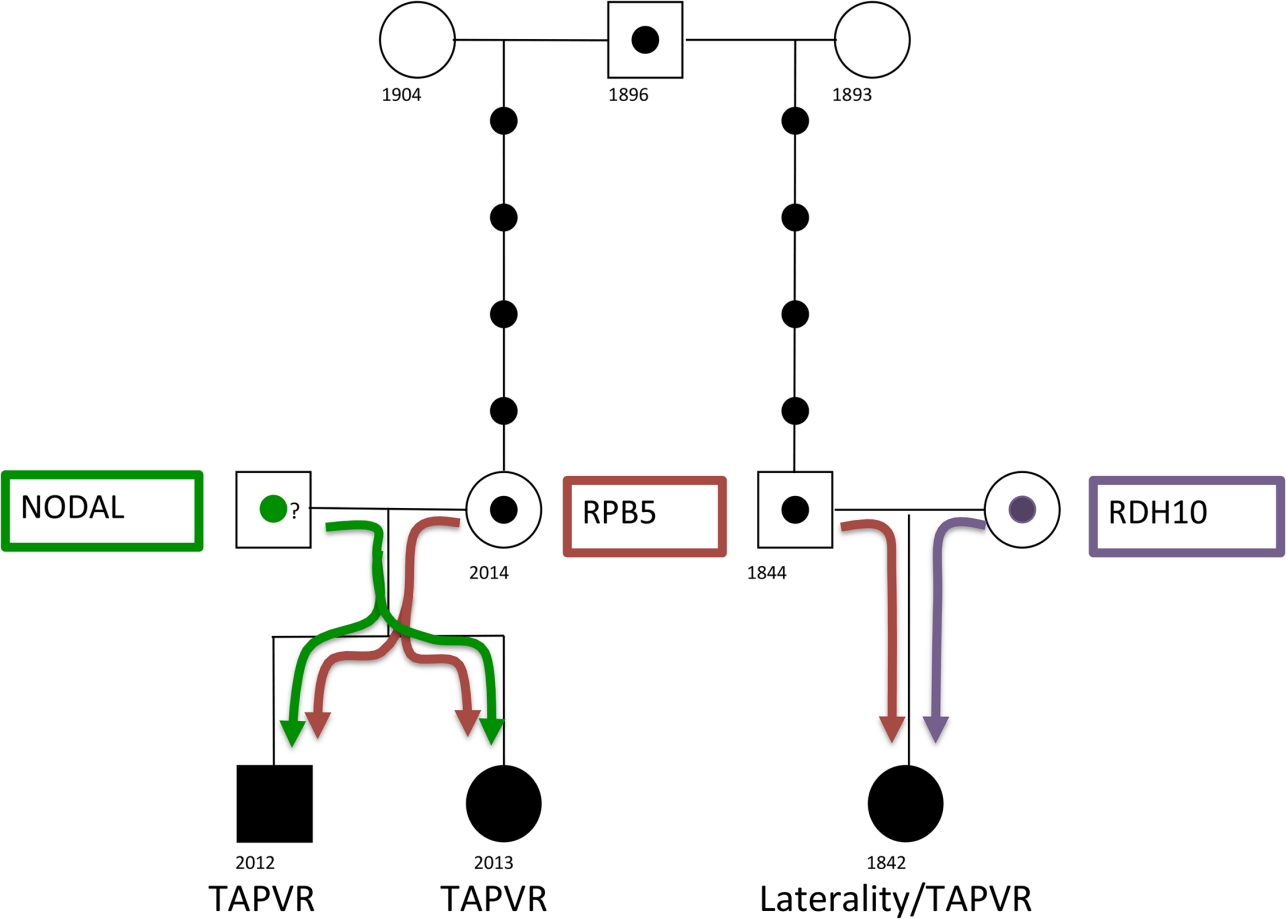
Model of digenic inheritence for TAPVR. The Rbp5 variant is inherited from a common ancestor whereas the “second genetic hits” (the Nodal and Rdh10 variants) are inherited from the other parent. Note DNA was not available for the father of patients 2012 and 2103. However, the NODAL variant was not detected in DNA isolated from the peripheral blood of their mother suggesting it was inherited from their father.

Based on our results, we propose a model where TAPVR occurs within this extended kindred due to combination of variants (at least two) in genes (Fig 8) involved in retinoic acid signaling. Consideration should also be given to environmental factors, specifically dietary intake of Vitamin A, as inadequate Vitamin A intake has been identified as a possible cause of CHD in native Canadians [24] and there is an increased incidence of TAPVR in this population [25].

In conclusion, we have presented several lines of evidence to support the claim that variants in the retinoic acid signaling pathway confer, at least in part, susceptibility to TAPVR. Through SGS mapping we dramatically narrowed the list of potential disease-causing variants obtained from whole genome sequencing and identified a damaging variant in the *retinol binding protein 5* (*RBP5*) gene that is overrepresented in TAPVR patients. In support of the significance of this variant as a TAPVR susceptibility gene we showed orthologous gene in zebrafish, *rbp7a*, is strongly expressed in the ciliated cells that establish LR patterning and that are upstream of the asymmetric expression of Nodal. Functional knockdown of *rbp7a* with a morpholino resulted in LR developmental defects. While this functional analysis awaits confirmation by generation of mutants of *rbp7a* in zebrafish due to the potential for spurious phenotypes observed in some morpholino models [26,27], the observation that a combination of subthreshold levels of rbp7a morpholino and *spaw* morpholino result in LR patterning defects implicates rbp7a and spaw in the same functional pathway, suggesting genetic interactions between the *retinol binding protein* and Nodal. This current approach reduces the levels of both rbp7a and Nodal/spaw, resulting in LR patterning defects that are not found in comparable reductions of either rbp7a or Nodal alone. The ideal modeling of the proposed mechanism of this multigenic human disease would be to use Crispr/cas9 technology in zebrafish to knock-in sequence variants analogous to the sequence variants found in our human genetics studies.

Further analysis of the whole genome data uncovered additional deleterious variants in retinoic signaling pathway members. Specifically, variants were detected in *NODAL* and *retinol dehydrogenase 10* (*RDH10*). Both of these variants were likely inherited from the parent not carrying the *RBP5* variant, suggesting digenic inheritance. These lines of evidence support genetic interactions between different members of the retinoic acid signaling pathway and begin to confirm the long-held notion that congenital heart defects are multifactorial conditions.

## Supporting Information

S1 Fig Alignment of human Rbp5 and zebrafish rbp7a protein sequences. Conserved amino acids are shown in red. The position of Glutamic acid (E) 70 is indicated by an arrow.
(TIF)

S1 Table Primers for genotyping of SNPs identified by WGS. (DOCX)

S2 Table Primers used for the PCR amplification of RBP5 and NODAL. (DOCX)
