# Supplementary figures and images for: Shared Segment Analysis and Next-Generation Sequencing Implicates the Retinoic Acid Signaling Pathway in Total Anomalous Pulmonary Venous Return (TAPVR)
Source: PLoS One. 2015 Jun 29;10(6):e0131514. doi: 10.1371/journal.pone.0131514 (PMC4485409; doi:10.1371/journal.pone.0131514)

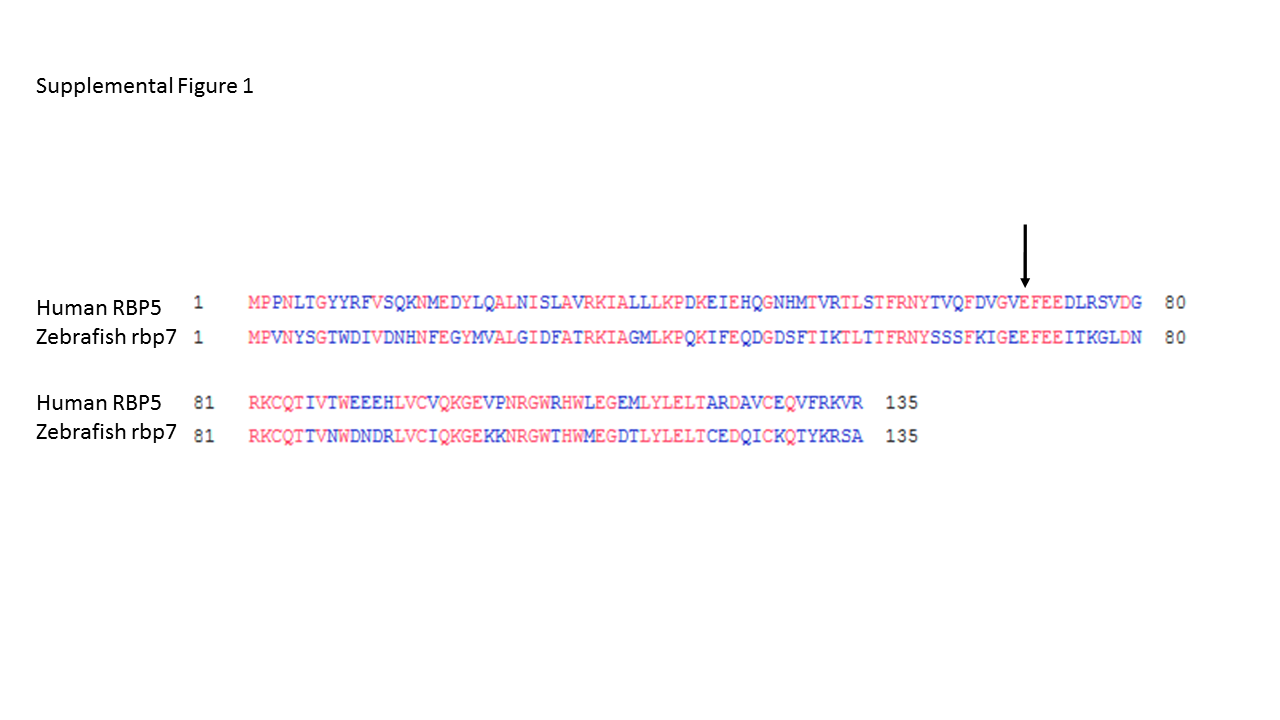

Supplement: S1 Fig — Conserved amino acids are shown in red. The position of Glutamic acid (E) 70 is indicated by an arrow. (TIF) [file pone.0131514.s001.TIF]
