# Supplementary material for: Shared Segment Analysis and Next-Generation Sequencing Implicates the Retinoic Acid Signaling Pathway in Total Anomalous Pulmonary Venous Return (TAPVR)
Source: PLoS One. 2015 Jun 29;10(6):e0131514. doi: 10.1371/journal.pone.0131514 (PMC4485409; doi:10.1371/journal.pone.0131514)
Supplement: S1 Table — (DOCX) [file pone.0131514.s002.docx]

| **Gene/**  **SNP ID** | **Forward Primer** | **Reverse Primer** | **Product Size (bp)** |
| --- | --- | --- | --- |
| NODAL/  rs150819707 | CTATTCTGACCTGCCCATCA | GGAGCACTCTGCCATTATCC | 162 |
| RBP5/  rs7969705 | AACCACATGACGGTGAGGAC | TCTCCTGTGATGCCTCCTTC | 160 |
| RDH10/  rs145171413 | TCGCCATGTGACTCACTTTC | CTCCTTGCGGACTCTTTCAG | 189 |

**S1 Table. Primers for genotyping of SNPs identified by WGS**
