# Supplementary material for: Shared Segment Analysis and Next-Generation Sequencing Implicates the Retinoic Acid Signaling Pathway in Total Anomalous Pulmonary Venous Return (TAPVR)
Source: PLoS One. 2015 Jun 29;10(6):e0131514. doi: 10.1371/journal.pone.0131514 (PMC4485409; doi:10.1371/journal.pone.0131514)
Supplement: S2 Table — (DOCX) [file pone.0131514.s003.docx]

**S2 Table. Primers used for the PCR amplification of RBP5 and NODAL**

| **Gene/**  **Exon** | **Forward Primer** | **Reverse Primer** | **Product Size (bp)** |
| --- | --- | --- | --- |
| **RBP5** |  |  |  |
| Exon 1 | GAGGGAAGAAGTCTGGTAGGG | GGGACCAAGAAGCAGGAAG | 334 |
| Exon 2 | CAGTCCCAGACCCAAAGAAG | GCTGGGGAAGGTCACTTTG | 375 |
| Exon 3 | CTGCTGTGGAGTAAAAGGGG | CTGGTTTGGACAAGGGGATG | 272 |
| Exon 4_1 | ACCTTGAGAAAGGGGCTCTG | CCCTTTGCCTGCTTCTTTC | 387 |
| Exon 4_2 | TGTTAATCTGTAACTTGCAGCCC | GTTGAACCCTGTGTGTGGG | 390 |
|  |  |  |  |
| **NODAL** |  |  |  |
| Exon 1 | CCCAGAGGGAGGAAAGGTG | ACTTCCCGAGTCCGCTG | 362 |
| Exon 2_1 | TGGTTGTGACACTGACTGAGG | TTCAGCCACTTGGAGAGAGG | 371 |
| Exon 2_2 | CAGCTGCTTAGAGCGGTTTC | CAAGTGATGTCGACGGTGC | 343 |
| Exon 2_3 | CAGAGGCAGCTGGGTGG | AGCAAAGCTAGAGCCCTGTC | 352 |
| Exon 3 | TTGCACTCAGGAACTGACTTTAAC | TTTGCCCCTCTCTGTTTCTC | 372 |
| RARE | CGCTAATCTGATCCGACCTG | GCACTCCAGCAAATTTCTCA | 175 |
